# LENGTH OF STAY IN U.S. AN ANOMALY: PREVENTIVE HEALTHCARE UTILIZATION AMONG FAITH-BASED FIRST-GENERATION CHINESE

**DOI:** 10.1093/geroni/igz038.1333

**Published:** 2019-11-08

**Authors:** Su-I Hou

**Affiliations:** The University of Central Florida, Orlando, Florida, United States

## Abstract

Limited knowledge exists on preventive healthcare services utilization (PHSU) among Chinese immigrants. This study examined factors related to PHSU among faith-based first-generation Chinese. A self-administered survey was conducted in five Chinese churches in southeastern US.(n=184). Mean age was 43 (SD=14) and 50% were males. Comparing with recent immigrants (<10 years), those who lived in the US for more than 10 years were more likely to be married (94.6% vs. 50.0%), have annual exams (74.5% vs. 39.6%), report doctors recommending cancer screenings (35.3% vs. 12.5%), have talked to doctors about screenings (25.5% vs. 4.2%), and perceive higher cancer knowledge (20.0% vs. 2.1%) (all p<.05). Considering all factors together, regressions showed perceived knowledge was the only significant predictor on having talked to doctors (OR=3.11) and doctor recommending screening (OR=2.15). Married status was the only strong predictor on annual exam (OR=29.13). Our findings highlight the urgent need for promoting PHSU in this population.

